# A revision of the genus *Doryodes* Guenée, 1857, with descriptions of six new species (Lepidoptera, Erebidae, Catocalinae, Euclidiini)

**DOI:** 10.3897/zookeys.527.6087

**Published:** 2015-10-15

**Authors:** J. Donald Lafontaine, J. Bolling Sullivan

**Affiliations:** 1 Research Associate, Canadian National Collection of Insects, Arachnids, and Nematodes, Biodiversity Program, Agriculture and Agri-Food Canada, KW Neatby Bldg., C.E.F., Ottawa, Ontario, Canada K1A 0C6; 2 200 Craven Street, Beaufort, North Carolina, USA 28516

**Keywords:** Taxonomy, Erebidae, Erebinae, Euclidiini, *Doryodes*, *Spartina*, *Aristida*

## Abstract

The genus *Doryodes* Guenée is revised to include ten species including six species described as new (*Doryodes desoto* Lafontaine & Sullivan; *Doryodes okaloosa* Sullivan & Lafontaine; *Doryodes fusselli* Sullivan & Lafontaine; *Doryodes reineckei* Sullivan & Lafontaine; *Doryodes broui* Lafontaine & Sullivan; and *Doryodes latistriga* Sullivan & Lafontaine). A key to species, descriptions, and illustrations of adults and genitalia are included.

## Introduction

The genus *Doryodes* Guenée, 1857, currently includes five species, four occurring in United States and Canada (Lafontaine and Schmidt 2010), and one occurring in the Bahamas.

Most species are very difficult to identify from external appearance, however, the vesica in males, the genitalia in females, and the barcodes, are diagnostic. All except one of the ten species in the genus are associated with coastal salt marshes.

## Materials and methods

### Repository abbreviations

Specimens were examined from the following collections:

BIO Biodiversity Institute of Ontario, University of Guelph, Ontario, Canada.

BMNH The Natural History Museum (statutorily, British Museum (Natural History), London, UK.

CNC Canadian National Collection of Insects, Arachnids, and Nematodes, Ottawa, Ontario, Canada.

EHMC Eric H. Metzler Collection, Alamogordo, New Mexico, USA.

FSCA Florida State Collection of Arthropods, McGuire Center for Lepidoptera & Biodiversity, Gainesville, Florida, USA.

JBSC J. Bolling Sullivan Collection, Beaufort, North Carolina, USA.

MEM Mississippi Entomological Museum, Mississippi State University, Mississippi, USA.

USNM National Museum of Natural History (formerly, United States National Museum), Washington, District of Columbia, USA.

VABC Vernon Antoine Brou Jr. Collection, Abita Springs, Louisiana, USA.

**Dissecting methods and genital terminology.** Dissection of genitalia and terms for genital structures and wing markings follow Lafontaine (2004).

## Systematics

### Doryodes

Taxon classificationAnimaliaLepidopteraErebidae

Guenée, 1857

#### Type species.

*Ligia acutaria* Herrich-Schäffer, [1852].

### Themma

Taxon classificationAnimaliaLepidopteraErebidae

Walker, 1863

#### Type species.

*Themma divisa*, 1863. Monotypy.

### Tunza

Taxon classificationAnimaliaLepidopteraErebidae

Waker, 1863

#### Type species.

*Tunza promptella* Walker, 183. Monotypy.

#### Diagnosis.

The genus *Doryodes* is easily recognized because of the elongated, apically pointed wings and the elongated abdomen. Males have broadly bipectinate antennae with pectinations 3–5 × as long as the width of the antennal shaft; females have filiform antennae. The frons is bare ventrally, covered with rough scaling dorsally. The eyes are rounded, without hairs or lashes. Ocelli are present. The labial palpus usually is directed forward, occasionally with the terminal segment angled ventrally; the basal and apical segments are about half as long as the middle segment. Forewing length varies from 13–21 mm, females, on average, are longer winged than males. The forewing in the male usually is pale buffy brown; some species have longitudinal streaks of gray, brown, or yellowish orange. There is a blackish-brown stripe along the middle of the wing, almost straight from the wing base to three-quarters of the distance to the apex, at which point it curves upward toward the apex and tends to narrow and fade out between the curve and the apex. The dark stripe is bordered dorsally by a narrow white line extending from the wing base to the point where the dark stripe curves upward; the dark stripe is bordered ventrally by a narrow white line on the outer half or third of the wing to, or almost to, the wing apex. Usually there are one or two dark dots representing the reniform spot, orbicular spot, or both. The forewing of the female usually is paler and more acutely-pointed apically than that of the male. The dark longitudinal stripe along the middle of the forewing is narrower in most females than in males. The hindwing is white to buff, and the color can vary with seasonal generations. In particular, many individuals captured from November to March are much darker than those found in spring and summer. The winter forms are often misidentified because superficially they differ more from the “summer” forms than most species differ from each other. The legs have spiniform setae on the tibia; the basitarsus has two or three ventral rows of spiniform setae. The abdomen is without tufts or abdominal brushes. In the male genitalia the tegumen, and especially the vinculum, are long and have a simple articulation with each other and with the valves; the vinculum ends ventrally as a deep V-shaped saccus. The uncus is shorter than the tegumen, bulbous at the base, then slightly tapered to the apex, with a spine-like tip. The juxta is strongly fused to the base of the valves, so it is difficult to spread the valves laterally without distorting or tearing the middle part of the genitalia. The valve is elongate and tripartite with the medial part lightly sclerotized apically. The base of the valve has a long lens-shaped sacculus extending about half way to the valve apex. The sacculus is extended as a sclerotized tube along (and fused with) the ventral margin of the valve, except toward the apex of the valve where the saccular extension is free from the valve and tapered to a pointed or blunt apex. A medial sclerotized ridge extends posteroventrally across the ventral surface of the valve from the base of the tegumen to fuse with the saccular extension on the lower edge of the valve (transverse ridge most prominent in *Doryodes tenuistriga*); the costal margin of the valve also is heavily sclerotized, more so toward the apex of the valve where it ends in a pointed or bluntly-rounded process, often free from the medial part of the valve at the apex. In one species (*Doryodes tenuistriga*) the sclerotized costal and ventral margins of the valves are broadly rounded at their apices and end well before the valve apex. The middle part of the valve is lightly sclerotized, especially the almost membranous, rounded, apical part of the valve. The aedeagus is elongated and cylindrical, 7–10 × as long as its mesial width in most species (4–5 × as long in *Doryodes tenuistriga* and *Doryodes okaloosa*). The vesica is about as long as the aedeagus in most species, although abruptly curved ventrally or laterally near its middle. The vesica has numerous diverticula, typically five (numbered on Figs 33, 34, 36, 37, 38, 39), each usually with a cornutus that may be broad and shaped like a shark fin, or slightly to markedly serrated into several to many basally-fused spines; the basal part of the vesica, just beyond the end of the aedeagus is often slightly to markedly swollen and has one to five sclerotized plates, often armed with one to many spinules. The narrow ductus seminalis arises near the middle of the vesica ventrally and typically clogs the narrow opening rather than everting during dissection. The recognition of most species requires examination of the relative position, size, and shape of the diverticula and their associated cornuti. The female genitalia are elongated with an oblong corpus bursae, which occasionally has a small signum, and has a well-developed and variably-sclerotized ventro-lateral appendix bursae posteriorly on the right. The ductus bursae is straight and varies in length, with the sclerotized plate wider at each end than mesially, except in *Doryodes reineckei* and *Doryodes tenuistriga*. The ductus bursae extends posteriorly as a sclerotized quadrangular plate over the ostium bursae. The ductus seminalis arises at the base of the appendix bursae posteriorly; in most species it is abruptly tapered at its base and thread-like after that, however in *Doryodes reineckei* and *Doryodes tenuistriga* it is wider and only gradually tapered. The anal papillae are lightly sclerotized and apically rounded or tapered, covered laterally with hair-like setae. The anterior apophyses are rod-like and usually are about as long as the posterior apophyses.

#### Distribution and biology.

Species of *Doryodes* occur from Atlantic Canada southward along the Atlantic Coast to the tip of peninsular Florida, and along the Gulf Coast to southern Texas and into Mexico. All species are associated with coastal salt marshes and creeks, except for *Doryodes bistrialis*, which occurs from North Carolina to Mississippi and Florida in pine savannas and other open habitats where wiregrass occurs. One species apparently is endemic to the Bahamas. Wagner et al. (2011) reared larvae of *Doryodes spadaria* in captivity on Bermuda grass (*Cynodon dactylon* (L.) Pers.), however, it is thought that species of cordgrass (*Spartina* spp.) are more probable larval hosts in salt marshes where it occurs. The larvae have longitudinal stripes typical of grass- and sedge-feeding species of Lepidoptera. *Doryodes bistrialis* is thought to feed on wiregrass (*Aristida stricta* Michx.).

#### Remarks.

Adults bear little superficial resemblance to other genera of the Erebinae: Euclidiini, however, the male genitalia are typical for the Euclidiini with the central apical part of the valve lightly sclerotized and rounded, and with the costal and ventral margins heavily sclerotized and apically free from the central part and extending into apical processes. The narrow-winged, longitudinally-streaked forewings of the adults are in keeping with the habitus of many grass and sedge-feeding species of Lepidoptera. All species for which data are available differ significantly in characters of the genitalia, particularly the shape of the vesica in males, and the shape of the bursa copulatrix in females, and also differ in the barcode sequences.

### Key to *Doryodes* based on adults and distribution

**Table:** 

1	Forewing brownish gray to whitish gray above and below the dark longitudinal medial stripe; Atlantic Canada and eastern United States	**2**
–	Forewing with orange stripe above and below dark longitudinal medial stripe; Bahamas	***Doryodes insularia***
2	Males	**3**
–	Females (females of two species from the Gulf Coast of Florida, *Doryodes desoto* and *Doryodes okaloosa*, are unknown)	**11**
3	Costal process of valve tapered to a point at or beyond central membranous part of valve	**4**
–	Costal processes of valve blunt and rounded apically and ending well before apical part of valve (Fig. 42); longitudinal stripe on forewing narrow and extending almost to wing apex; Gulf Coast of Texas and Louisiana	***Doryodes tenuistriga***
4	Vesica with diverticulum 1 with deeply-serrated rooster-comb-like sclerotized cornutus (Figs 33–36, 41)	**5**
–	Vesica with diverticulum 1 with triangular shark-fin-like sclerotized cornutus, which may have minute serrations on one side (Figs 37–40)	**9**
5	Vesica with diverticulum 1 elongated, 2–5 × as long as mesial width (Figs 33, 34)	**6**
–	Vesica with diverticulum 1 about as long as mesial width (Figs 35, 36, 41)	**7**
6	Diverticulum 1 usually 2–3 × as long as mesial width; diverticulum 2 on posterior surface; apex of vesica symmetrical with broad triangular cornutus on each side projecting laterally (Fig. 33); inland species associated with wiregrass savannah from North Carolina to Mississippi and southern Florida	***Doryodes bistrialis***
–	Diverticulum 1 4–5 × as long as mesial width; diverticulum 2 lateral on left; apex of vesica asymmetrical with single triangular cornutus on diverticulum 5 (Fig. 34); salt marsh species known from Gulf Coast of Florida	***Doryodes desoto***
7	Aedeagus short, about 5 × as long as wide (Fig. 35); ventral process on valve broadly rounded apically; Gulf Coast of Florida	***Doryodes okaloosa***
–	Aedeagus 7–10 × as long as wide (Figs 36, 41); ventral process on valve tapered apically	**8**
8	Diverticulum 2 of vesica larger than diverticulum 1 (Fig. 36); forewing with longitudinal stripe dark brown and sharply defined; known only from coastal North Carolina; superficially not safely distinguishable from *Doryodes spadaria*, although usually smaller	***Doryodes fusselli***
–	Diverticulum 2 of vesica a slight hump (Fig. 41); forewing with longitudinal stripe faint and diffuse, fading into paler coloration below stripe; Gulf Coast from the Florida Panhandle to Texas	***Doryodes reineckei***
9	Vesica T- or Y-shaped at apex with diverticula 4 and 5 projecting to each side, each with a triangular cornutus at apex (Figs 37, 38); Atlantic Coast from Canada to southern Florida and on southwestern coast of Florida	***Doryodes spadaria***
–	Vesica with rounded, bulging apex with preapical cornuti on left side (Figs 39, 40); Gulf Coast from Alabama to Texas	**10**
10	Longitudinal forewing stripe broad, sharply defined or diffuse; vesica with position of diverticulum 1 barely raised from curve in vesica; apex of vesica an elongated lobe (Fig. 39)	***Doryodes latistriga***
–	Longitudinal forewing stripe narrow; diverticulum 1 of vesica a prominent posterior lobe from curve of vesica; apex of vesica bluntly rounded (Fig. 40)	***Doryodes broui***
11	Sclerotized plate in ductus bursae about as long as posterior width of plate; left posterior side of corpus bursae with protruding sclerotized lobe opposite appendix bursae; ductus bursae and corpus bursae very short (Fig. 49)	***Doryodes tenuistriga***
–	Sclerotized plate in ductus bursae about 2–4 × as long as posterior width (Figs 43–48); left posterior side of corpus bursae tapered opposite appendix bursae	**12**
12	Forewing mainly white; longitudinal dark stripe absent or barely discernable; sclerotized plate in ductus bursae broad posteriorly, tapered anteriorly, 0.55–0.65 × length of ductus bursae (Fig. 48)	***Doryodes reineckei***
–	Forewing pale buffy brown with dark longitudinal stripe sharply defined; sclerotized plate in ductus bursae broader anteriorly and posteriorly, narrower in middle, 0.75–0.85 × length of ductus bursae (Figs 43–47)	**13**
13	Corpus bursae slightly constricted mesially; appendix bursae elongated; sclerotized plate in ductus bursae not abruptly widened at anterior end (Figs 43, 45)	**14**
–	Corpus bursae constricted post-mesially at base of rounded, wrinkled appendix bursae; sclerotized plate in ductus bursae widened at anterior end (Figs 44, 46, 47)	**15**
14	Ductus bursae tapered from posterior end to anterior end; appendix bursae rounded posteriorly (Fig. 43); inland species of pine savannah associated with wiregrass; North Carolina to Florida and Mississippi	***Doryodes bistrialis***
–	Ductus bursae almost even in width throughout; appendix bursae truncated posteriorly (Fig. 45); salt march species known only from coastal North Carolina	***Doryodes fusselli***
15	Longitudinal forewing stripe broad, sharply defined or diffuse (Figs 16, 17); appendix bursae with posterior margin straight and heavily sclerotized (Fig. 46)	***Doryodes latistriga***
–	Longitudinal forewing stripe narrow, sharply defined (Figs 8, 22); appendix bursae rounded or notched posteriorly and lightly sclerotized (Figs 44, 47)	**16**
16	Sclerotized plate over ostium bursae deeply concave on posterior margin; appendix bursae bilobed posteriorly (Fig. 44); Atlantic Coast from Canada to southern Florida and on Gulf Coast as far north as Punta Gorda	***Doryodes spadaria***
–	Posterior margin of ostium bursae essentially straight (Fig. 47); Gulf Coast from Alabama to Texas	***Doryodes broui***

**Figures 1–8.**
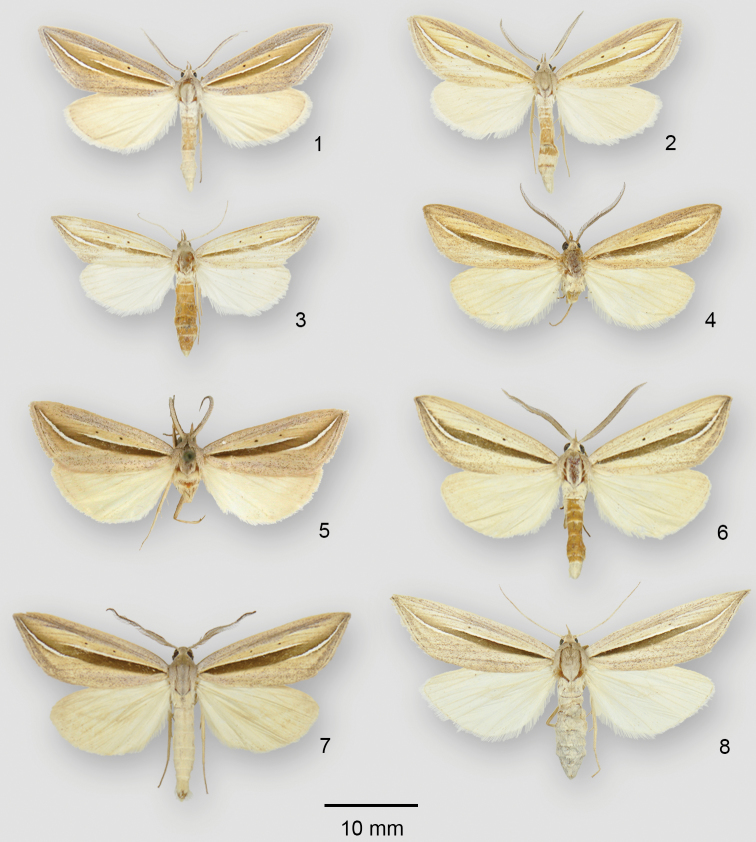
*Doryodes* adults. **1** *Doryodes bistrialis* ♂, USA, Florida, Marion Co., Ocala Nat’l Forest FR-88 **2** *Doryodes bistrialis* ♂, USA, North Carolina, Pender Co., Holly Shelter Game Land **3** *Doryodes bistrialis* ♀, USA, North Carolina, Pender Co., Holly Shelter Game Land **4** *Doryodes desoto* holotype ♂, USA, Florida, Pinellas Co., Ft Desoto Park nr St. Petersburg **5** *Doryodes okaloosa* holotype ♂, USA, Florida, Okaloosa Co., Shalimar **6** *Doryodes spadaria* ♂, USA, North Carolina, Onslow Co., Camp Lejeune, Corn Landing **7** *Doryodes spadaria* ♂, USA, South Carolina, Charleston Co., The Wedge Plantation, 7 mi N McClellanville **8** *Doryodes spadaria* ♀, USA, South Carolina, Charleston Co., The Wedge Plantation, 7 mi N McClellanville.

**Figures 9–16.**
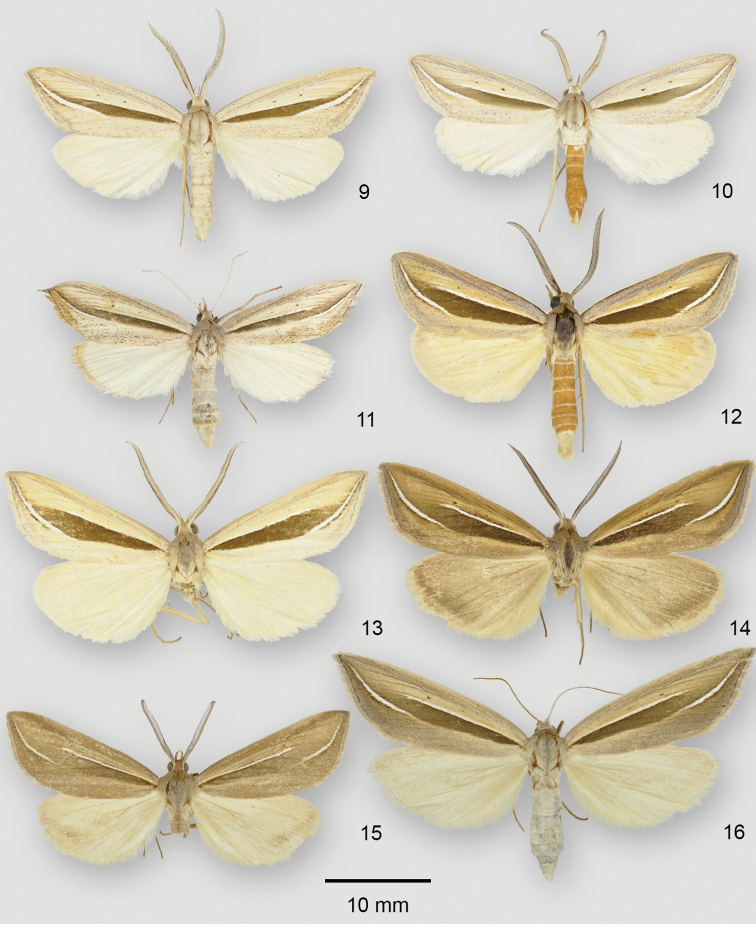
*Doryodes* adults. **9** *Doryodes spadaria* ♂, USA, North Carolina, Carteret Co., Ft Macon State Park **10** *Doryodes fusselli* paratype ♂, USA, North Carolina, Carteret Co., Ft Macon State Park **11** *Doryodes fusselli* paratype ♀, USA, North Carolina, New Hanover Co., Fort Fisher Maritime Forest **12** *Doryodes latistriga* paratype ♂, USA, Alabama, Baldwin Co., Camp Beckwith **13** *Doryodes latistriga* paratype ♂, USA, Mississippi, Harrison Co., Long Beach **14** *Doryodes latistriga* paratype ♂, USA, Louisiana, Lafourche Parish, nr Golden Meadow **15** *Doryodes latistriga* paratype ♂, USA, Louisiana, St Tammany Parish, 4.2 mi N Abita Springs **16** *Doryodes latistriga* paratype ♀, USA, Mississippi, Jackson Co., Gulf Islands Nat. Seashore.

**Figures 17–24.**
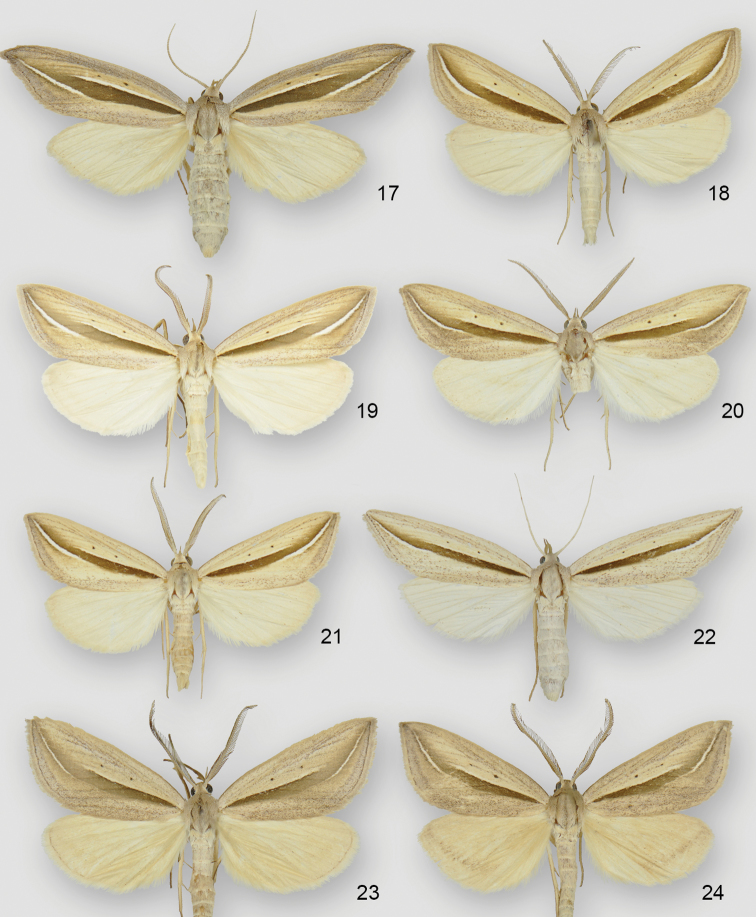
*Doryodes* adults. **17** *Doryodes latistriga* paratype ♀, USA, Mississippi, Harrison Co., Long Beach **18** *Doryodes broui* holotype ♂, USA, Louisiana, St John Parish, Edgard **19** *Doryodes broui* paratype ♂, USA, Mississippi, Harrison Co., Long Beach **20** *Doryodes broui* paratype ♂, USA, Texas, Jackson Co., Deutschburg **21** *Doryodes broui* paratype ♂, USA, Louisiana, St John Parish, Edgard **22** *Doryodes broui* Paratype ♀, USA, Louisiana, St Tammany Parish, 4.2 mi N Abita Springs **23** *Doryodes reineckei* paratype ♂, USA, Louisiana, St Tammany Parish, 4.2 mi N Abita Springs **24** *Doryodes reineckei* paratype ♂, USA, Louisiana, St Tammany Parish, 4.2 mi N Abita Springs.

**Figures 25–32.**
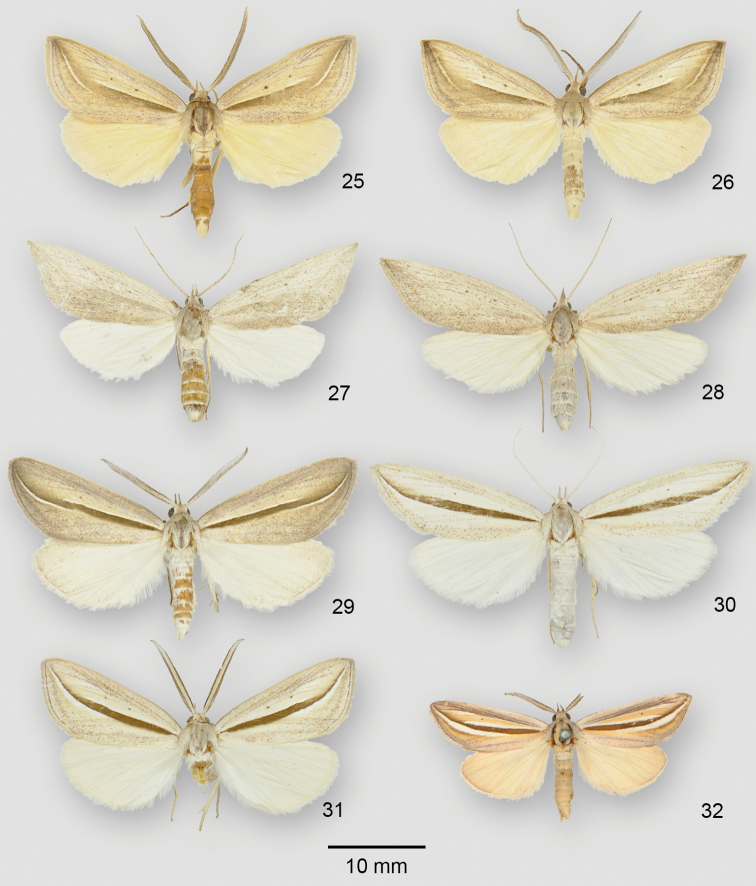
*Doryodes* adults. **25** *Doryodes reineckei* paratype ♂, USA, Alabama, Baldwin Co., Camp Beckwith **26** *Doryodes reineckei* paratype ♂, USA, Alabama, Baldwin Co., Camp Beckwith **27** *Doryodes reineckei* paratype ♀, USA, Louisiana, St Tammany Parish, 4.2 mi N Abita Springs **28** *Doryodes reineckei* paratype ♀, USA, Louisiana, St Tammany Parish, 4.2 mi N Abita Springs **29** *Doryodes tenuistriga* ♂, USA, Texas, Cameron Co., Laguna Atascosa **30** *Doryodes tenuistriga* ♀, USA, Texas, Cameron Co., Brownsville. **31** *Doryodes tenuistriga* ♂, USA, Louisiana, Cameron Parish, Johnson Bayou. **32** *Doryodes insularia* syntype ♂, Bahamas, Nassau.

**Figures 33–36.**
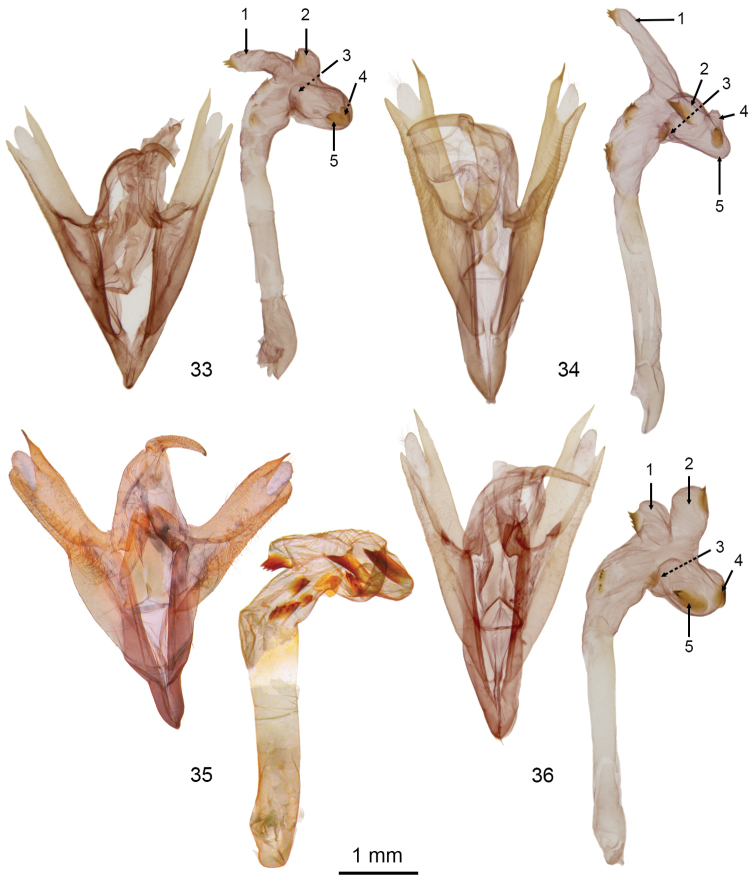
*Doryodes* male genitalia (vesica from left lateral view). **33** *Doryodes bistrialis*, Florida, Stemper, CNC slide 16409 **34** *Doryodes desoto* holotype, CNC slide 16050 **35** *Doryodes okaloosa* holotype, JBS slide FLMNH-MGCL 02951 **36** *Doryodes fusselli* paratype, CNC slide 16418.

**Figures 37, 38.**
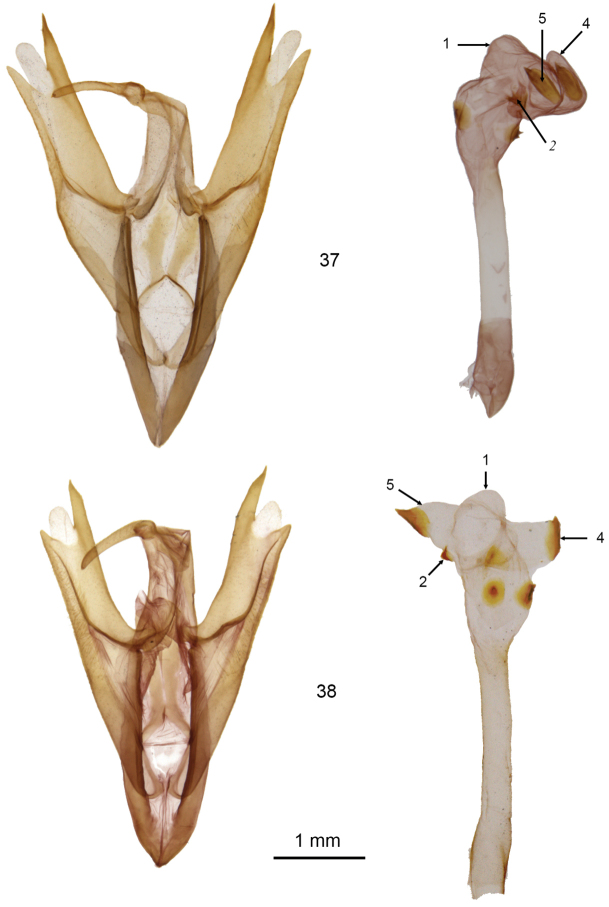
*Doryodes* male genitalia. **37** *Doryodes spadaria* (vesica from left lateral view), CNC slide 16051 (valves), 16415 (vesica) **38** *Doryodes spadaria* (vesica from ventral view), CNC slide 16415 (valves), 16052 (vesica).

**Figures 39–42.**
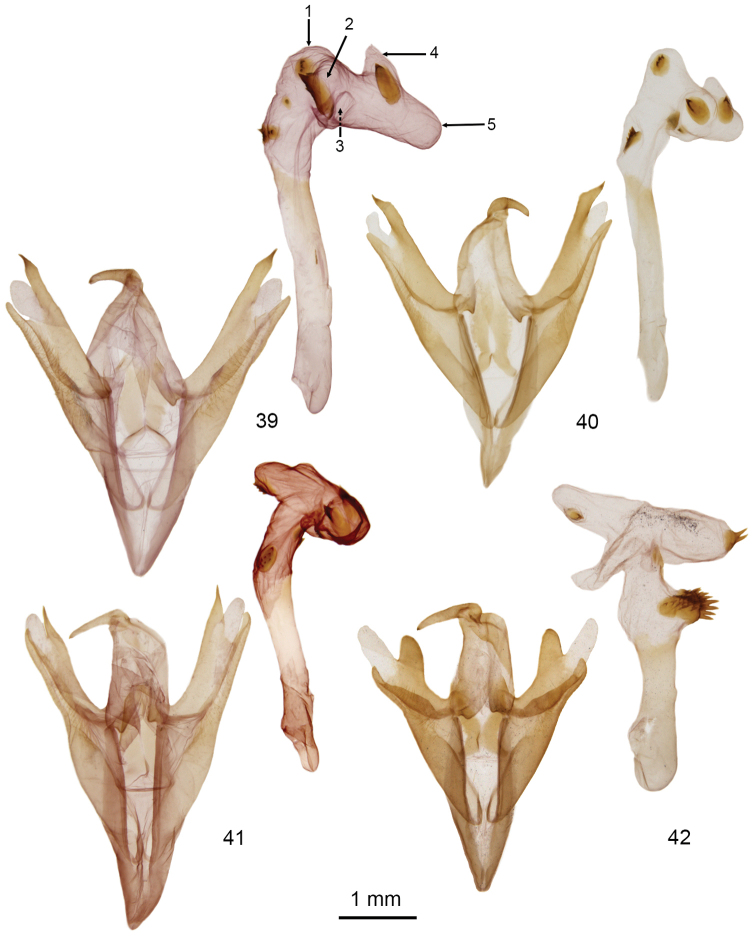
*Doryodes* male genitalia (vesica from left lateral view). **39** *Doryodes latistriga* paratype, CNC slide 16049 **40** *Doryodes broui* holotype, CNC slide 16053 **41** *Doryodes reineckei* paratype, CNC slide 16414 **42** *Doryodes tenuistriga*, CNC slide 16055.

**Figures 43–49.**
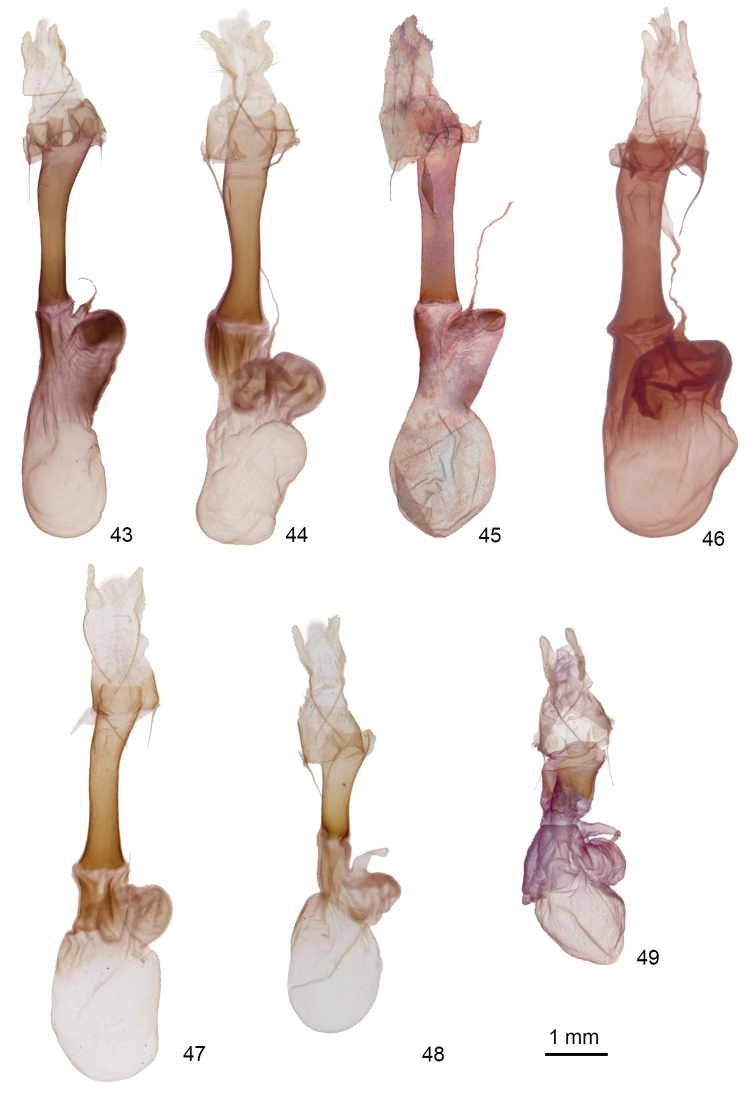
*Doryodes* female genitalia **43** *Doryodes bistrialis*, CNC slide 16046 **44** *Doryodes spadaria*, CNC slide 16047 **45** *Doryodes fusselli*, JBS-6645 **46** *Doryodes latistriga* paratype, CNC slide 16439 **47** *Doryodes broui* paratype, CNC slide 16057 **48** *Doryodes reineckei* paratype, CNC slide 16056 **49** *Doryodes tenuistriga*, CNC slide 16798.

### *Doryodes* Guenée, 1857

#### Doryodes bistrialis

Taxon classificationAnimaliaLepidopteraErebidae

(Geyer, 1832)

Figs 1–3 , 33 , 43

Agriphila bistrialis Geyer, 1832: 38; pl. 133, figs 775, 776.
Ligia acutaria Herrich-Schäffer, [1852]: 74; pl. 73, fig. 447.

##### Type material.

*Agriphila bistrialis*: type lost. Given the difficulty of identifying species of *Doryodes* from an illustration, and the lack of a type locality for *Agriphila bistrialis*, we designate a neotype in order to preserve the long-standing identity of the inland species of *Doryodes*. **Neotype** ♂, USA, North Carolina, Carteret Co., Croatan National Forest, Sam Hatcher Road, 23 April 2006, J. Bolling Sullivan. BOLD barcode Sample ID: 06-NCCC-932 [USNM].

*Ligia acutaria* is nominally described from southern Russia, although already in 1852 Herrich-Schäffer suspected it was mislabeled. The type is lost, so to ensure that the current synonymy is maintained, we designate the neotype of *Agriphila bistrialis* as **Neotype** of *Ligia acutaria* also.

##### Other material examined and distribution.

We have examined material from North Carolina east of the Piedmont, from most of Florida except for the Keys and Panhandle and a single male from a power cut near Grand Bay National Wildlife Refuge, Jackson County, Mississippi. Specimens from Florida, Mississippi and North Carolina are closely similar in appearance, genitalia and barcodes.

##### Diagnosis.

Superficially, adults usually can be identified by the narrower dark stripe on the forewing and their relatively small size (forewing length: ♂ 13.0–15.5 mm, ♀ 14.5–16.0 mm). Compared to *Doryodes spadaria* the medial longitudinal stripe on the forewing of *Doryodes bistrialis* is much narrower and the hindwings are whitish not buff through June emergences. Later in the year, *Doryodes spadaria* hindwings become more whitish, so wing length and the width of the longitudinal line must be relied upon to distinguish the species, or genital characters must be examined for positive identification. Females of *Doryodes bistrialis* do not overlap those of *Doryodes spadaria* in size, females of *Doryodes spadaria* having a forewing length of 18.0–21.0 mm. The male vesica is also diagnostic in *Doryodes bistrialis* in that there is a line of smaller cornuti extending along the trunk usually in three patches, whereas in *Doryodes spadaria* there are two side-by-side cornuti on the basal trunk of the vesica. In *Doryodes bistrialis*, diverticulum 1 is 2–3 × as long as wide and has a deeply-serrated rooster-comb-like cornutus at the apex; *Doryodes spadaria* has no diverticulum in this position and the cornuti in the vesica are triangular, sometimes minutely serrated on one side. *Doryodes bistrialis* can be distinguished from *Doryodes fusselli* by the shape of diverticulum 1, which is rounded in *Doryodes fusselli* and about as long as wide; also the apex of the vesica in *Doryodes bistrialis* is symmetrical with a triangular cornutus on a pouch on each side (diverticula 4 and 5). *Doryodes bistrialis* can be distinguished from *Doryodes reineckei* and *Doryodes latistriga* by the wing pattern and by size and from *Doryodes broui* by the characters of the vesica. Along the Gulf Coast *Doryodes bistrialis* could be confused with *Doryodes broui* and *Doryodes tenuistriga*, but characters of the vesica (*Doryodes broui*, Fig. 40 and *Doryodes tenuistriga*, Fig. 42, versus *Doryodes bistrialis*, Fig. 33) readily distinguish these three species and *Doryodes bistrialis* occurs farther inland. The female genitalia of *Doryodes bistrialis* are elongated with a more compact, less differentiated, appendix bursae than in *Doryodes spadaria*.

##### Distribution and biology.

*Doryodes bistrialis*, unlike all other species in the genus, occurs mainly inland away from coastal salt marshes. It occurs in pine savannas where wiregrass (*Aristida stricta*), the presumed food plant, is abundant. It has only been recorded in North Carolina, Mississippi and Florida, and it follows the distribution of the presumed foodplant, *Aristida stricta*. The species is on the wing from April through October in North Carolina, and throughout the year in Florida. The species appears to be associated with wiregrass, but we were unable to successfully rear it on cut grasses. Eggs should be placed on potted *Aristida* and larvae monitored to determine their preference for the grass or detritus in the base of the grass clump. In North Carolina the savannas are usually a half mile or more inland from coastal marshes and extend westward into the Sandhills adjacent to the piedmont. It is possible that the salt marsh species and the wiregrass species could occur in the same or very close areas where coastal marshes penetrate inland but we did not find such areas.

#### Doryodes desoto

Taxon classificationAnimaliaLepidopteraErebidae

Lafontaine & Sullivan sp. n.

http://zoobank.org/16419019-B413-405D-99A9-8717D8C12BF1

Figs 4 , 34

##### Type material.

**Holotype** ♂. **Florida**, Pinellas Co., Ft. Desoto Park nr St. Petersburg, 4 Jan. 1968, J. D. Lafontaine. CNC. **Paratypes**: 2 ♂. **Florida**. Gulf Co., Rd. to Cape San Blas, nr Port St Joe, 29.8°N, 85.3°W, 31 July 1998, Jeff Slotten, genitalia slide FLMNH-MGCL 02944 (1 ♂). Florida, Sarasota Co., Siesta Key, 3 Feb. 1952, C.P. Kimball, genitalia slide FLMNH-MGCL 02948 (1 ♂). FSCA.

##### Etymology.

This species is named after Ft. De Soto Park, Florida.

##### Diagnosis.

This species is superficially indistinguishable from *Doryodes spadaria*, *Doryodes fusselli*, and *Doryodes broui*, although its range on the Gulf Coast of Florida is north and west of the range of *Doryodes spadaria* and *Doryodes fusselli*, and east of the range of *Doryodes broui*. Forewing length is 16 mm, on the small size for *Doryodes spadaria*, but within the normal range of the other similar species. The species can be definitively identified only by the male vesica, particularly by the long, narrow diverticulum 1, which is about 4–5 × as long as its basal width and with an apical, deeply serrated rooster-comb-like cornutus; diverticulum 2 is on the left side of the vesica, not posterior as in *Doryodes bistrialis*; diverticulum 4 is preapical on the right, without a cornutus, and diverticulum 5 forms a projecting lobe with a cone-shaped diverticulum on its left side.

##### Description.

External structural characters as described for genus. Forewing length 16 mm; forewing whitish buff with slightly darker-buff and pale-gray streaks; a prominent blackish-brown stripe along the middle of wing, curving upward and tapered at about ¾ from base; stripe narrower than for *Doryodes spadaria* and *Doryodes okaloosa*, but wider than for *Doryodes bistrialis*; stripe bordered by narrow white line above extending to ¾ from base, and with similar white line below stripe extending from above forewing tornus almost to wing apex. Hind wing white with very faint buffy tone. Male genitalia mainly as described for genus. Dorsal heavily-sclerotized margin of valve extending beyond middle membranous part, then tapered abruptly into a sharp spine; ventral sclerotized margin of valve not evenly tapered, but widens slightly preapically then tapered to blunt point free from inner membranous part of valve. Aedeagus cylindrical, 8–9 × as long as mesial width. Vesica with swollen area distal to end of aedeagus, 0.4–0.5 × as long as aedeagus and about 2 × as long as wide, with two spinule-covered sclerotized plates, these partially or completely divided into as many as four plates each with less spinules; diverticulum 1 finger-like, 4.5–5.5 × as long as mesial width, with apical serrated cornutus; diverticulum 2 rounded, on left side at base of diverticulum 1, with shark-fin-like cornutus; diverticulum 3 quadrate, on right side and without a cornutus; diverticulum 4 preapical, on right side without a cornutus; diverticulum 5 forming a bulbous apical lobe with a rounded spine-tipped cornutus on left side.

##### Distribution and biology.

This species is known only from three male specimens, all from the Gulf Coast of Florida between Sarasota County and Gulf County. Collecting dates are in January, February, and July. Nothing is known of its biology except it is associated with coastal salt marshes.

#### Doryodes okaloosa

Taxon classificationAnimaliaLepidopteraErebidae

Sullivan & Lafontaine sp. n.

http://zoobank.org/1CA76187-9C9D-4972-865C-B0CBB4D6A6A6

Figs 5 , 35

##### Type material.

**Holotype** ♂, **Florida**, Okaloosa Co., Shalimar, black light trap, 3461, H. G. Hilton, genitalia slide FLMNH-MGCL 02951. FSCA.

##### Etymology.

The species name is in honor of the Okaloosa, a tribe of the Creek Nation and longtime inhabitants of the area.

##### Diagnosis.

This species probably occurs with *Doryodes desoto* and *Doryodes reineckei* in the salt marshes and tidal creeks throughout the coastal panhandle area of Florida. The species is slightly larger than *Doryodes bistrialis*, which may occur nearby but inland. The washed out appearance of *Doryodes reineickei* immediately distinguishes it from *Doryodes okaloosa*. *Doryodes desoto* is very similar and at present dissection of the male genitalia is the only reliable way to distinguish these two species, although based on the single specimen of *Doryodes okaloosa*, it appears that *Doryodes okaloosa* is broader winged than *Doryodes desoto*, but not as broad winged as *Doryodes reineckei*. The abundance of large cornuti in the vesica, as well as the spatulate lower process of the valve and the short, broad aedeagus distinguishes *Doryodes okaloosa* from all other *Doryodes* species.

##### Description.

Forewing length 16.5 mm; forewing stripe dark brown, narrower than for *Doryodes spadaria* but wider than for *Doryodes desoto*; forewing wider and browner than in *Doryodes spadaria* and *Doryodes desoto*, but not as wide as in *Doryodes reineckei*, and longitudinal stripe sharply defined, unlike that of *Doryodes reineckei*. Antennae missing but presumed to be bipectinate as in other species in genus. Hind wing white with buff tinge. Male genitalia mainly as described for genus. Dorsal heavily-sclerotized margin of valve wider and less well defined than in other species except for tapered spine-like apex; apex not rounded as in *Doryodes tenuistriga*, but wider than in other species; ventral sclerotized margin of valve slightly tapered to a broadly spatulate apex, much as in *Doryodes tenuistriga*. Aedeagus cylindrical, about 5 × as long as mesial width. Vesica with swollen area distal to end of aedeagus about 0.5 × as long as aedeagus and about 2 × as long as wide, with four large spine-covered sclerotized plates; diverticulum 1 slightly longer than wide with large deeply-serrated cornutus near apex; three preapical diverticula, each with large shark-fin-like cornutus; arrangement of large cornuti not seen in any other species of *Doryodes*.

##### Distribution and biology.

At present this species is known from the holotype collected in Okaloosa County, Florida. It likely ranges south in the coastal brackish marshes toward the St. Petersburg/Tampa area and to the west along the Florida coast but little collecting has been done in salt marshes of the Florida Panhandle.

#### Doryodes spadaria

Taxon classificationAnimaliaLepidopteraErebidae

Guenée, 1857

Figs 6–8 , 9 , 37 , 38 , 44

Doryodes spadaria Guenée, 1857: 234.
Themma divisa Walker, 1863: 186.
Tunza promptella Walker, 1863: 196.
Doryodes spadaria race grandipennis Barnes & McDunnough, 1918: 117; pl. 17, figs 1, 2, **syn. rev.**

##### Type material.

***Doryodes spadaria***: type lost. It is impossible to identify this species from the description given by Guenée, but the type, listed as being from Florida, would most likely be from the East Coast, which would mean it represented the species now known either as *Doryodes bistrialis*, *Doryodes fusselli* or *Doryodes spadaria*. In order to maintain the longstanding identity of this species as the widespread species in salt marshes of the Atlantic Coast, we designate the lectotype of *Themma divisa* Walker as **neotype** of *Doryodes spadaria*, an action that will ensure the current identity and synonymy. The data are given under *Themma divisa*. ***Themma divisa*: lectotype** ♂, here designated, BMNH, examined; male in good condition except right antenna and tip of left antenna missing; forewing length 18.1 mm; data “E. Doubleday, St John’s Bluff, E Florida/ Themma divisa/ syn-type [blue circle]/ 40-1-14-84/ S.O. 153.” ***Tunza promptella*: holotype** ♂, no locality, BMNH, examined. **Doryodes spadaria race grandipennis: syntypes** ♂, ♀, USNM, examined.

##### Other material examined and distribution.

Canada: Quebec, Nova Scotia, Prince Edward Island. USA: Maine, New York, New Jersey, Maryland, North Carolina, South Carolina, Georgia, Florida.

##### Diagnosis.

External structural characters as described for genus. *Doryodes spadaria* is the most widespread and common species in the genus, and except for *Doryodes fusselli* in coastal North Carolina, all specimens of *Doryodes* from salt marshes along the Atlantic Coast of Canada and the United States that we have seen are *Doryodes spadaria*. Adults are relatively larger (forewing length: ♂ 13–20 mm, most commonly 16–18 mm; ♀ 18–21 mm, most commonly 19 mm) than those of *Doryodes bistrialis* (forewing length: ♂, 13.5–15.5 mm, ♀ 14.5–16.0 mm), the species most likely to be confused with it in southeastern United States outside of North Carolina where the smaller *Doryodes fusselli* also occurs in salt marshes. The forewing ground color in males varies from whitish buff to yellow buff with gray streaks; the longitudinal dark stripe is dark brown and conspicuously wider than in *Doryodes bistrialis*; the ground color in females averages paler than that of males and the wings and dark longitudinal stripe are narrower. In the male genitalia, most structural characters are as described for the genus; the sclerotized costal margin of the valve is more heavily sclerotized apically and extends farther beyond the central membranous part of the valve than in most other species; the sclerotized ventral margin of the valve ends in a blunt point before the end of the central part of the valve. Two examples of the genitalia are illustrated, one to show the typical orientation of the vesica in comparison with other species (Fig. 37), and a ventral orientation of the vesica (Fig. 38) to illustrate the shape and positions of the two preapical diverticula and their cornuti. The vesica is slightly shorter than the aedeagus; the basal, swollen part of the vesica is armed with two thorn-like cornuti on sclerotized plates; the left preapical diverticulum (# 5) is tapered to a large triangular cornutus, whereas the right preapical diverticulum (# 4) has a bulge in the middle and a smaller cornutus. In the female genitalia the corpus bursae is elongated with the part anterior to the opening of the ductus bursae swollen laterally and with longitudinal “ribbons” of sclerotization. The appendix bursae is lightly sclerotized and slightly bilobed posteriorly. The ductus bursae is almost as long as the corpus bursae, and is heavily sclerotized dorsally and ventrally with the plates slightly narrower mesially and expanded laterally and more heavily sclerotized at their junction with the corpus bursae; the ventral plate at the end of the ductus bursae is extended posteriorly as a quadrangular plate that projects over the opening to the ductus with the posterior margin of the plate concave. The anterior and posterior apophyses are similar in length (posterior slightly longer), about half the length of the ductus. The anal papillae are lightly sclerotized, produced ventrally anteriorly, rounded posteriorly, with the surface setose.

##### Distribution and biology.

*Doryodes spadaria* is widely distributed in coastal salt marshes on the Atlantic Coast of Canada and the United States from eastern Quebec to southern Florida. A few inland records in southern Florida (e.g., Kissimmee Prairie) are anomalous, unless there is brackish water in these areas. Adults occur in Canada and northeastern United States from June to August. In southeastern United States there are spring and summer generations and at least three generations in Florida.

#### Doryodes fusselli

Taxon classificationAnimaliaLepidopteraErebidae

Sullivan & Lafontaine sp. n.

http://zoobank.org/AF95A06B-AFB2-4CDD-B51C-18540F87E766

Figs 10 , 11 , 36 , 45

##### Type material.

**Holotype** ♂, **North Carolina**, New Hanover Co., Fort Fisher Maritime Forest, A trail, 775504 W 335833 N, 15 Watt UV trap, Coastal fringe Evergreen Forest, June 3, 1995, J. Bolling Sullivan, Richard Broadwell & Brad Smith. USNM. **Paratypes**: 8 ♂, 4 ♀. **North Carolina**. Same data as for holotype (2 ♂, 2 ♀); same locality and collector as holotype, June 27, 1995 (1 ♂, 1 ♀), April 19, 1995 (1 ♂), November 8, 1994 (1 ♂), October 5, 1994 (1 ♀). North Carolina, Carteret Co., Ft Macon State Park, 15 July 1999 (1 ♂); Carteret Co., maritime scrub, N34.697°, W-76.683°, 29 Aug. 2005, J. Bolling Sullivan, barcodes 05-NCCC-523, & 05-NCCC-524 (2 ♂). BIO, CNC, JBSC, USNM.

##### Etymology.

The species name is in honor of John Fussell from Morehead City, North Carolina, who has worked tirelessly for decades to describe and protect the unique flora and fauna of the North Carolina coastal plain, particularly the Croatan National Forest. All of our lives are richer for his efforts.

##### Diagnosis.

This species occurs with *Doryodes spadaria* in the salt marshes and tidal creeks throughout coastal North Carolina Adults are slightly larger than *Doryodes bistrialis*, but noticeably smaller (especially females) than *Doryodes spadaria*. The medial chocolate stripe on the forewing is broader than in *Doryodes bistrialis*, but narrower than that of *Doryodes spadaria*. Spring males are larger than those of the summer and fall generations, so they are more easily confused with *Doryodes spadaria*. The hind wing is white, without the buff coloring of *Doryodes spadaria*; in late summer some males of *Doryodes spadaria* can have white hind wings, but size ranges for the two species do not overlap in this generation. Similar changes in size with season are seen for many other species (Sullivan and Miller 2007). The vesica differs in having a diffuse line of cornuti along the basal trunk and by the symmetrical pair of cornuti-tipped diverticula at the apex. *Doryodes spadaria* is described in full, so only differences from it are given in the description.

##### Description.

Smaller than *Doryodes spadaria*; spring males (forewing length: 16–17 mm [16–20 mm for *Doryodes spadaria*]), slightly larger than summer males (forewing length: 14–15 mm); some small individuals (forewing length: 12–13 mm) late in season; females of *Doryodes fusselli* (forewing length: 16 mm) smaller than those of *Doryodes spadaria* (forewing length: 18–21 mm) but larger than those of *Doryodes bistrialis*. Hindwing pearly white. Male genitalia with valves similar to those of *Doryodes spadaria* but slightly smaller (valve length: 4.45 mm versus 4.75 mm for *Doryodes spadaria*). Vesica basal trunk with single band of cornuti, usually separated into two elongated sections; diverticulum 1 rounded with toothed, rooster-comb-like cornutus on dorsal side; diverticulum 2 rounded, about 1.0–1.5 × as long as wide, with triangular cornutus on ventral surface near apex; terminus of vesica with two similar cornuti-tipped diverticula projecting distally [in *Doryodes spadaria* two terminal diverticula are more elongated, different in shape from each other, and project laterally]. Female genitalia differ from those of *Doryodes spadaria* in more even width of ductus bursae, and appendix bursae more elongated with posterior margin even, not bilobed as in *Doryodes spadaria*.

##### Distribution and biology.

At present this species is known only from North Carolina, occurring from Dare County in the north to Brunswick and New Hanover counties in the south. It is likely that it occurs farther south but may have been overlooked as *Doryodes spadaria*. Specimens have been collected from April through October and the species appears to be on the wing continuously. Eggs were obtained from a female and fed cut *Spartina alterniflora* Loisel. leaves and fresh and wilted Bermuda grass. The larvae survived to the second instar and were similar to those of *Doryodes spadaria* (Wagner et al. 2011). Larvae have not been located in the field.

#### Doryodes latistriga

Taxon classificationAnimaliaLepidopteraErebidae

Sullivan & Lafontaine sp. n.

http://zoobank.org/7DF697E9-B84B-4B13-9257-640523BB3568

Figs 12–16 , 17 , 39 , 46

##### Type material.

**Holotype** ♂, **Alabama**. Baldwin Co., Camp Beckwith, UV trap, *Spartina*-*Juncus* marsh, N 30.39538°; W -87.84657°; 5 Aug. 2009. J. Bolling Sullivan. USNM. **Paratypes**: 44 ♂, 9 ♀. **Alabama**. Same data as holotype (2 ♂); same locality and collector as holotype, 7, 9 & 14 August 2009 (3 ♂); Baldwin Co., Weeks Bay Preserve, N 30.414°, W -87.833°; 4 Aug. 2009. J. Bolling Sullivan, barcodes 09-MISC-046, 047, 048, 049 (4 ♂). **Louisiana**. St. Tammany Parish, 4.2 mi NE Abita Springs, sec 24, T6S R12E, N 30°30.986’, W 89°57.276’, 25 Feb. 1997, V.A. Brou Jr. CNC slide ♂ 16049 (1 ♂); same locality and collector, 20 May & 27 Nov. 1983 (2 ♂), 23 May 1984 (1 ♂), 26 March 1985 (1 ♂), 27 March 1987 (1 ♂), 14 Aug. 1988 (1 ♂), 13 Jan. & 20 March 1989 (2 ♂), 13 Sept. 1998 (1 ♂), 28 May 2000 (1 ♂), 12 Oct. 2001 (1 ♂), 27 Jan. & 16 March 2002 (2 ♂), 3 April 2008, Barcode CNCLEP00113508 (1 ♂), 11 April 2008 (1 ♂), 12 April & 28 Oct. 2009 (2 ♂, 1 ♀), 21 March 2010 (1 ♂), 7 Feb. 2011, CNC slide 16058 (1 ♀), 22 April 2012, Barcode CNCLEP00113566) (1 ♂). Cameron Parish, Johnson’s Bayou, 16 March 2002, V.A Brou Jr. (1 ♀). Lafourche Parish, near Golden Meadow, 5 March 2006, V.A. Brou Jr., CNC slide ♂ 16054 (1 ♂); same locality and collector, 2 July 2005 (1 ♀), 5 March 2006 (6 ♂). **Mississippi**. Harrison County, Long Beach, 17 June 1992, V.A. Brou Jr. (1 ♀); same locality, 2 June 1996, CNC slide ♂ 16679, R. Kergosien (1 ♂), same locality and collector, 2 May 1997, CNC slide ♂ 16681 (1 ♂), 20 Nov. 1992, CNC slide ♂ 16706 (1 ♂), 17 May 1996, CNC slide ♀ 16440, (1 ♀). Jackson County, Gulf Coast Islands National Seashore, 19–20 April 1985, R.L. Brown (1 ♀). Jackson Co., Ocean Springs, 12, 15, 17 June 1992 & 1 Aug. 1992 (5 ♂, 2 ♀), V.A. Brou Jr. CNC, JBSC, MEM, USNM, VABC.

##### Etymology.

The name of this species refers to the width of the longitudinal dark stripe on the forewing in both sexes.

##### Other material examined and distribution.

We have examined material from southern Alabama, Mississippi, and Louisiana. Specimens in Bold database exhibit considerable heterogeneity but all sequences are within 0.8% of each other.

##### Diagnosis.

This species occurs with *Doryodes broui*, *Doryodes reineckei*, and *Doryodes tenuistriga*. It is the largest species and with the most distinctive forewing pattern. It should not be confused with *Doryodes tenuistriga*, which has a narrow forewing stripe, or with *Doryodes broui*, which is smaller (forewing length: 13.0–15.5 mm) and also has a narrower forewing stripe. It can be distinguished from *Doryodes reineckei* by the breadth and distinctness of the longitudinal stripe; in females of *Doryodes reineckei* the forewing stripe is absent or pale gray, hardly contrasting with the ground color. The most distinguishing character of the male genitalia is the elongated terminal diverticulum of the vesica that projects ventrally. Females are similar to males but have longer, more pointed forewings and whiter hindwings. The female genitalia are the most robust of any *Doryodes* species. The corpus bursae has a bulge on the right side that is more pointed than in the other species, and the appendix bursae is quadrate with the posterior margin almost straight and heavily sclerotized.

##### Description.

Forewing ground color in spring and summer specimens yellowish white to buff with gray streaking, hindwing white to whitish buff; forewing in fall and winter specimens darker with more brown shading, hindwing variably suffused with brown, especially along wing margin. Forewing length 14.5–18.0 mm (males), 17.5–20.0 mm (females), similar in length to those of *Doryodes spadaria*, but wings somewhat broader. Longitudinal stripe broader than in any other species in genus. Male genitalia with cornuti in variable patches on basal trunk of vesica, larger patch dorsal and most basal, two smaller patches of cornuti lateral and more distal (but one or both can be absent). Vesica with diverticulum 1 absent, its position represented by rounded curve of vesica posterior to swollen part of vesica after aedeagus; diverticulum 2 a rounded bulge on left side of vesica with large, slightly-serrated shark-fin-like cornutus; diverticula 3 a short quadrate pouch on right side almost opposite position of diverticulum 2; diverticulum 4 cone shaped, dorsolaterally on right, with bulbous spine-tipped cornutus at base near junction with elongated ventral lobed representing diverticulum 5. Female genitalia similar to those of the other species but more robust, and appendix bursae quadrate with posterior margin almost straight and heavily sclerotized.

##### Distribution and biology.

The adults are found in tidal creeks and salt marshes from Alabama to Louisiana. The biology is unknown, but presumed to be similar to other species of *Doryodes* that occur in similar habitats. Adults occur throughout the year, but concentration of collecting dates suggests a primary brood from March to May and a secondary brood in September and October (V.A. Brou Jr. pers. comm.).

#### Doryodes broui

Taxon classificationAnimaliaLepidopteraErebidae

Lafontaine & Sullivan sp. n.

http://zoobank.org/B53AB087-6A59-436F-B0CB-91DA7C90172F

Figs 18–22 , 40 , 47

##### Type material.

**Holotype** ♂, **Louisiana**. St. Tammany Parish, 4.2 mi NE Abita Springs, sec 24, T6S R12E, N 30°30.986’, W 89°57.276’, 3 May 2010, V.A. Brou Jr.; slide CNC 16053; barcode CNCLEP 00113565. CNC. **Paratypes**: 98 ♂, 49 ♀. **Louisiana**. Same locality and collector as holotype, 25 April 1984, CNC slide ♀ 16057 (1 ♀), 24 May 1984 (1 ♀), 25 April 1985 (1 ♂), 5 & 28 May & 30 June 1986 (3 ♂), 2, 15 & 16 June & 12 Aug. 1988 (4 ♂), 5 & 20 March & 28 April 1989 (4 ♀), 16 & 22 June & 20 Dec. 1990 (2 ♂, 1 ♀), 14 April 1991 (1 ♀), 22 May & 1 Nov. 1992 (1 ♂, 1 ♀), 18 Oct. 1993 (1 ♀), 11 & 29 May, 13 June & 16 Sept. 1996 (4 ♂), 1 March & 9 April 1997 (2 ♂), 26 & 28 May 1998 (2 ♂), 5, 8 & 11 May 1999 (2 ♂, 1 ♀), 26 Jan. & 15 May 2000 (1 ♂, 1 ♀), 18 & 19 March 2002 (1 ♂, 2 ♀), 5, 11 & 31 May 2003 (3 ♂, 2 ♀), 3 June 2005 (1 ♀), 19 May & 2 & 12 June 2006 (2 ♂, 1 ♀), 20 Feb., 6 April, & 6 & 25 May 2008 (2 ♂, 2 ♀), 26 March & 12 May 2009 (2 ♂), 21 March & 13 May 2010 (1 ♂, 3 ♀), 7 & 23 April, 3, 21 & 25 May & 7 July 2011 (2 ♂, 4 ♀), 8 & 10 April & 12 May 2013 (1 ♂, 3 ♀). St. Tammany Parish, Hwy 90 at Hwy 433, 8 May 1971, E.H. Metzler, Female genitalia on slide # E.H.M. 686 (1 ♀). Cameron Parish, Johnson’s Bayou, 19 April 1985, CNC slide ♀ 16048 (1 ♂, 1 ♀), 10 & 19 Sept. 1985, (4 ♂), 14 Sept. 1990, V.A. Brou Jr. (3 ♂). Cameron Parish, Little Chenier, 14 May 1981, V.A. Brou Jr. (1 ♂). Lafourche Parish, Golden Meadow, 28 April 1975, V.A. Brou Jr. (2 ♂). Lafourche Parish, near Golden Meadow, 25 March 2007, V.A. Brou Jr. (1 ♀). St. John the Baptist Parish, Edgard, 29 March, 2 & 9 April & 9 June 1976 (4 ♂, 1 ♀), 7 April, 20 May, 5 June & 5 Aug. 1977 (4 ♂), 10 Aug., 6 May, 1 June & 7 Aug. 1978 (4 ♂, 1 ♀), 1 April, 12 & 21 May, 24 July & 17 Aug. 1979 (3 ♂, 3 ♀), 25 June, 25 July, 4 & 12 Aug., 27 Sept., 17 & 29 Oct. & 2 Dec. 1980 (7 ♂, 2 ♀), 17 April, 9 May, 4 & 6 June & 2 Oct. 1981 (6 ♂, 1 ♀), 16 & 25 March, 17 & 20 April, 12, 21 & 24 May, 4 & 26 June, 11 Aug., 6 Sept., 7 Oct. & 25 Dec. 1982 (11 ♂, 7 ♀), 14 May 1983 (1 ♂), 23 May 1984 (1 ♀), V.A. Brou Jr. St. John the Baptist Parish, Edgard, 10 May 1971, E.H. Metzler, Male genitalia on slide # E.H.M. 685 (1 ♂). Vermillion Parish, Intracoastal City, 26 July 1984, V.A. Brou Jr. (1 ♂). **Mississippi**. Harrison County, Long Beach, 18 May 1992, R. Kergosien (1 ♂); same collector and locality, 15 May 1997, CNC slide 16680 (1 ♂); Jackson Co., Grand Bay Nat’l Wildlife Refuge, N 30°41.3’ W 60°40.6’, coastal marsh savanna, 21 July 2014, J. Bolling Sullivan, barcodes 14-NCCC-470, 471, 472, 473, 474 & 475 (5 ♂). **Texas**. Jackson Co., Deutechburg, 7 Oct. 1974, A. & M.E. Blanchard, slide CNC 16682 (1 ♂); Brownsville, 6-11, Geo. Dorner (1 ♂). CNC, EHMC, JBSC, MEM, USNM, VABC.

##### Etymology.

We name this species after Vernon A. Brou, Jr. in recognition of his impressive and tireless efforts in collecting and researching the Lepidoptera of Louisiana.

##### Diagnosis.

This species occurs with *Doryodes latistriga*, *Doryodes reineckei*, and *Doryodes tenuistriga* in coastal salt marsh habitats from Alabama to southern Texas. *Doryodes broui* is superficially indistinguishable from *Doryodes spadaria*, but differs from it in male genitalia, barcodes, and occurs far to the west of the known range of *Doryodes spadaria*. It can be distinguished from *Doryodes reineckei* in having a sharply-defined longitudinal dark stripe on the forewing, and from *Doryodes latistriga* by the narrower forewing stripe in *Doryodes broui*. It is most likely to be confused with *Doryodes tenuistriga*, which typically is larger, but because of variation in both species, some specimens must be dissected or barcoded, for positive identification. The male genitalia of *Doryodes broui* are most similar to those of *Doryodes latistriga*, but the diverticula and cornuti are smaller, especially the terminal diverticulum (# 5), which is short and rounded, not elongated as in *Doryodes latistriga*.

##### Description.

Forewing length: 13.0–15.5 mm (males), 13.5–17.0 mm (females), Forewing buffy brown to whitish gray with faint buffy streaks, darker forms in colder months; longitudinal stripe dark brown, similar in width to that of *Doryodes spadaria*, narrower than for *Doryodes latistriga*, wider than for *Doryodes tenuistriga*. Male genitalia. Aedeagus 8 × as long as mesial width; vesica with dorsolateral toothed triangular cornutus on left side of basal part of vesica distal to end of aedeagus; a sclerotized plate in anterior 90° bend in vesica at position of ductus seminalis; posterior curve in vesica extended posteriorly into rounded diverticulum 1 with toothed preapical cornutus; diverticulum 2 on left side reduced to low bulge with large conical cornutus in middle; preapical posterior diverticulum (# 4) tapered with conical cornutus at distal base and also at base of short rounded apical diverticulum 5. The female genitalia are similar to those of *Doryodes latistriga*, but the appendix bursae is rounded posteriorly and only lightly sclerotized.

##### Distribution and biology.

*Doryodes broui* occurs from Alabama to southern Texas. Nothing is known of its biology. Adults occur throughout the year, but concentration of collecting dates suggests a primary brood between mid-March and mid-June and a secondary protracted brood between late July and mid-October (V.A. Brou Jr. pers. comm.).

#### Doryodes reineckei

Taxon classificationAnimaliaLepidopteraErebidae

Sullivan & Lafontaine sp. n.

http://zoobank.org/07D8C57D-B2CF-478B-84A5-5642E4BA09D9

Figs 23–24 , 25–28 , 41 , 48

##### Type material.

**Holotype** ♂, **Alabama**. Baldwin Co., Camp Beckwith, UV trap, Spartina-Juncus marsh, W 30.39538; N -87.84657; 7 Aug. 2009. J. Bolling Sullivan. USNM. **Paratypes**: 122 ♂, 74 ♀. **Alabama**. Same data as for holotype but collected 5 Aug. 2009 (one with slide CNC 16414) (5 ♂), 9 Aug. 2009, one with barcode label CNCLEP 00113509 (2 ♂); Baldwin Co., Weeks Bay Preserve, N 30.414°, W -87.833°; 4 Aug. 2009. J. Bolling Sullivan, barcodes 09-MISC-050, 051, 052, 053 (4 ♂). **Louisiana**. St. Tammany Parish, 4.2 mi NE Abita Springs, sec 24, T6S R12E, N 30°30.986’, W 89°57.276’, 8 April 1983, V.A. Brou Jr. (1 ♂); same locality and collector, 15 & 20 May, 2 Sept. 1983 (5 ♂, 2 ♀), 13, 26–30 April, 8 & 23 May & 8 Aug. 1984 (2 ♂, 7 ♀), 26 & 28 April 1985 (1 ♂, 1 ♀), 1 May, 10 May, CNC ♀ slide 16056, 11, 14, 16 & 22 May 1986 (5 ♂, 4 ♀), 9 & 31 May & 29 Nov. 1988 (2 ♂, 1 ♀), 8 April, 25 May & 6 June 1989 (2 ♂,1 ♀), 26 Feb., 10 March, 14 May & 2 June 1990 (3 ♂, 1 ♀), 12 April 1991 (1 ♀), 24 June 1992 (1 ♂), 14 April & 16 Nov. 1993 (2 ♂), 17 April & 4 May 1994 (2 ♂), 29 March, 23 April & 2 Aug. 1995 (3 ♂), 20 & 22 April, 20 May & 29 May 1996 (4 ♂, 2 ♀), 24 Feb. & 1 March 1997 (2 ♂), 16 June 1998 (1 ♂), 4 April 1999 (1 ♂), 19 March 2000 (2 ♀), 28 April 2001 (1 ♂), 24 Jan., 15, 16 & 22 March 2002 (3 ♂, 7 ♀), 7 & 8 April 2005 (1 ♂, 2 ♀), 27 March, 26 April & 2 May 2006 (3 ♂), 28 April, 5 May & 6 June 2007 (3 ♂), 28 March slide CNC 16410 ♂, 31 March, 11 April barcode CNCLEP 00113564, 16 April, 1 May, 19 June & 11 Aug. 2008 (4 ♂, 3 ♀), 14 Feb., 21 March, 12, 19, 21 & 28 April 2009 (7 ♂, 1 ♀), 21 March, 3, 23 & 25 April, 5, 24 & 26 May 2010 (6 ♂, 2 ♀), 21 Jan., 8, 22, 25, 26 & 27 April, 3, 8 & 28 May 2011 (8 ♂, 6 ♀), 12 Feb., 10 April 2012, barcodes CNCLEP 00113563 & 00113568, 22 April, 9 June 2012 (2 ♂, 3 ♀), 10 April 2013, barcode CNCLEP 00113511, 10 April 2013, CNC slide ♀ 16436 (2 ♀), V.A. Brou Jr. St. Tammany Parish, Hwy 90 at Hwy 433, 8 May 1971, E.H. Metzler, Female genitalia on slide # E.H.M. 683 (1 ♀). Cameron Parish, Johnson’s Bayou, 23 Oct. 1985, V.A. Brou Jr. (1 ♂). Lafourche Parish, Cut Off, 21 Feb., 11 & 19, 20, 21, & 25 April, 16 May 1975, V.A. Brou Jr. (3 ♂, 14 ♀). Lafourche Parish, near Golden Meadow, 2 July & 6 Aug. 2005 (3 ♀), 25 March 2007, V.A. Brou Jr. (1 ♀). St. John the Baptist Parish, Edgard, 23 April 1973, (1 ♂), 16 April 1975 (1 ♂), 26 March, 18 & 23 April & 1 May 1976 (5 ♂, 1 ♀), 22 April, 5, 6 & 23 May 1977 (3 ♂, 2 ♀), 1, 7, 8 & 11 May & 3 June 1978 (5 ♂), 22 March, 1, 13 & 24 April & 2 May 1979 (6 ♂, 2 ♀), 9 May 1981 (1 ♂), 6 Jan., 16 & 24 March, 2, 17, 18 & 24 April 1982 (6 ♂, 1 ♀), 14 May 1983 (2 ♂, 1♀), V.A. Brou Jr. **Mississippi**. Harrison County, Long Beach, 11 Feb. 1995, R. Kergosien (1 ♂); Jackson Co., G.C.R.L. Ocean Sp. [Gulf Coast Research Laboratory, Ocean Springs], 25 January 1993, R. Kergosien (1 ♂). CNC, EHMC, FSCA, JBSC, MEM, USNM, VABC.

##### Etymology.

The species is named for John P. Reinecke, a retired USDA entomologist who worked in Mississippi and developed insect organ culture techniques and detailed the anatomy of the hindgut of larval Lepidoptera.

##### Diagnosis.

This species occurs with *Doryodes latistriga*, *Doryodes broui*, and *Doryodes tenuistriga* in coastal salt marsh habitats from Alabama to Texas. *Doryodes bistrialis* may occur nearby on the Gulf Coast, but inhabits inland longleaf pine savannas rather than salt marshes. *Doryodes reineckei* is immediately distinguished from other species of *Doryodes* in having a broader forewing in the male, the wing pattern appears faded and smudged, and the ventral margin of the longitudinal stripe appears to blend into the forewing ground color below it, not sharply defined as in other species. The male genitalia of *Doryodes reineckei* are smaller than those of *Doryodes latistriga*, but the characters of the vesica are definitive. In *Doryodes reineckei* the three patches of cornuti on the basal trunk of the vesica are rotated to the left, so the basal one is more lateral than dorsal; diverticulum 1 of the vesica projects dorsally and has a rooster-comb-like cornutus, whereas in *Doryodes latistriga* and *Doryodes broui* there is a tapered diverticulum (or rounded lobe) projecting posteriorly with a small spined cornutus on the side, and diverticulum 2 has no cornutus in *Doryodes reineckei*, whereas in the other two species there is a large cornutus at the base of the diverticulum. Females are immediately distinguishable by their whitish-gray forewing color with the longitudinal stripe is absent, or very faint, compared with the normal pattern seen in other species of *Doryodes*. Females have acutely pointed forewings.

##### Description.

Forewing length 15.0–17.0 mm (males), 17.0–20.0 mm (females). Male noticeably broader winged than other species of *Doryodes*. Forewing pale brown to dark gray brown, darker forms in colder months; longitudinal dark stripe paler and less sharply defined than in other species, lower margin of stripe blending into darker ground color below it; wing margin more rounded than in other species. Male genitalia with valve similar in size to that of *Doryodes spadaria* but aedeagus much shorter (2.29 versus 2.97 mm), about 7.5 × as long as mesial width; basal trunk of the vesica with three patches of cornuti, two ventrolaterally on right side of aedeagus, largest one basal, dorsolaterally on left with multiple spinules on a heavily-sclerotized oval plate. Vesica above basal trunk T-shaped, elongated posteriorly-directed diverticulum 1 with a preapical rooster-comb-like cornutus on anterior surface, a small rounded diverticulum 2 on posterior right side opposite end of aedeagus, a sclerotized plate at 90° ventral angle in vesica next to ductus seminalis, and a ventral rounded apical diverticulum 5 with a conical cornutus on left side. Female genitalia disproportionally small, especially sclerotized plate in ventral wall of ductus bursae only 2.5 × as long as posterior width, plate tapered anteriorly, only 0.55–0.65 × as long as ductus bursae; ductus seminalis broad at base and gradually tapered, so appearing much wider than other species except *Doryodes tenuistriga*, and, like *Doryodes tenuistriga*, corpus bursae more rounded than in other species; appendix bursae a rounded lightly-sclerotized lobe with slightly bilobed posterior margin.

##### Distribution and biology.

The species has been collected from the western panhandle of Florida along the Gulf Coast to eastern Texas. Dates are from April to August but it is likely on the wing throughout the year. Nothing is known of its biology other than its association with *Spartina* marshes. Adults probably occur throughout the year, but most records are from a large brood occurring between mid-March and late June, with a minor second brood in the late summer and fall (V.A. Brou Jr. pers. comm.).

#### Doryodes tenuistriga

Taxon classificationAnimaliaLepidopteraErebidae

Barnes & McDunnough, 1918

Figs 29–31 , 42 , 49

Doryodes tenuistriga Barnes & McDunnough, 1918: 117.

##### Type material.

**Syntypes** ♂, ♀, Benito, Texas, USNM, examined.

##### Other material examined and distribution.

**USA**: Louisiana, Texas.

##### Diagnosis.

*Doryodes tenuistriga* adults usually can be distinguished from other species in the genus by the narrow forewing stripes. The forewing length varies from 16.5–18.0 mm (males) and 15.5–18.5 mm (females). Some specimens of *Doryodes broui* can be similar, but the male and female genitalia are diagnostic. In the male genitalia the sclerotized areas on the dorsal and ventral margins of the valve end in broadly rounded processes well before the mainly membranous apex of the valve; in other species the process on the dorsal margin of the valve is pointed and extends to the apex of the valve, or beyond it, and the ventral process extends to, or almost to, the valve apex and is bluntly pointed or narrowly rounded. The aedeagus is short and wide, only about 5 × as long as wide. In the vesica the dorsal and ventral diverticula are similarly elongated, giving the vesica a T-shape; the vesica immediately posterior to the aedeagus has a rounded ventral lobe with a massive spine-covered sclerotized apical plate that is unique in the genus. In the female genitalia the ventral sclerotized plate on the ductus bursae is short and wide, about as long as wide and extending about ½ length of ductus; corpus bursae rounded, with protruding sclerotized lobe on posterior left opposite appendix bursae; appendix bursae rounded, lightly sclerotized.

##### Distribution and biology.

*Doryodes tenuistriga* is known only from the Gulf Coast of Texas and Louisiana, occurring as far south as Brownsville, Texas. Its range overlaps those of *Doryodes broui*, *Doryodes reineckei*, and *Doryodes latistriga*, and among these species, it is likely only to be confused with *Doryodes broui*. The immature stages and larval host plants are unknown. Based on very few records, it appears that *Doryodes tenuistriga* flies throughout the year but with a primary brood in April and May and a secondary protracted brood in the fall (V.A. Brou Jr. pers. comm.).

#### Doryodes insularia

Taxon classificationAnimaliaLepidopteraErebidae

Hampson, 1904

Fig. 32

Doryodes insularia Hampson, 1904: 174.

##### Type material.

**Syntype** ♂, Nassau, Bahamas, BMNH, examined.

**Diagnosis.** *Doryodes insularia* is unique in the genus because of small size (forewing length: 12.5 mm), the white lines bordering the longitudinal dark stripe on the forewing are thicker than those of any other species, there is a contrasting orange-brown band below the forewing costa and another one below the white line bordering the lower margin of the black stripe, and occurrence in the Bahamas. The species is known only from the type series. The type material in the Natural History Museum, London, has not been dissected.

##### Distribution and biology.

Bahamas. Nothing is known of the biology of *Doryodes insularia*.

#### Check list

930925 *Doryodes bistrialis* (Geyer, 1832)

syn. *Doryodes acutaria* (Herrich-Schäffer, [1852])

930925.1 *Doryodes desoto* Lafontaine & Sullivan, 2015

930925.2 *Doryodes okaloosa* Sullivan & Lafontaine, 2015

930927 *Doryodes spadaria* Guenée, 1857

syn. *Doryodes divisa* (Walker, 1863)

syn. *Doryodes promptella* (Walker, 1863)

syn. Doryodes spadaria race grandipennis Barnes & McDunnough, 1918

930927.1 *Doryodes fusselli* Sullivan & Lafontaine, 2015

930927.2 *Doryodes latistriga* Sullivan & Lafontaine, 2015

930927.3 *Doryodes broui* Lafontaine & Sullivan, 2015

930927.4 *Doryodes reineckei* Sullivan & Lafontaine, 2015

930928 *Doryodes tenuistriga* Barnes & McDunnough, 1918

* *Doryodes insularia* Hampson, 1904 (*Bahamas)

## Supplementary Material

XML Treatment for Doryodes

XML Treatment for Themma

XML Treatment for Tunza

XML Treatment for Doryodes bistrialis

XML Treatment for Doryodes desoto

XML Treatment for Doryodes okaloosa

XML Treatment for Doryodes spadaria

XML Treatment for Doryodes fusselli

XML Treatment for Doryodes latistriga

XML Treatment for Doryodes broui

XML Treatment for Doryodes reineckei

XML Treatment for Doryodes tenuistriga

XML Treatment for Doryodes insularia
